# Preparation of nanofibrous poly (L-lactic acid) scaffolds using the thermally induced phase separation technique in dioxane/polyethylene glycol solution

**DOI:** 10.1080/15685551.2023.2194175

**Published:** 2023-03-26

**Authors:** Tao Zhang, Kangyao Chen, Xing Wu, Xiufeng Xiao

**Affiliations:** a Department of Orthopedics Institute, Fuzhou Second Hospital of Xiamen University, Fuzhou China; b Department of Orthopedics Institute,The Third Clinical Medical College, Fujian Medical University, Fuzhou; c Department of Orthopedics, Fuzhou Second Hospital of Xiamen University, China; d Fujian Provincial Key Laboratory of Advanced Materials Oriented Chemical Engineering, College of Chemistry and Material Science, Fujian Normal University, Fuzhou, China

**Keywords:** Poly (L-lactic acid), polyethylene glycol, nanofibrous, phase separation

## Abstract

Porous nanofibrous poly (L-lactic acid) (PLLA) scaffolds were fabricated in combination with a thermally induced phase separation technique using a dioxane/polyethylene glycol (PEG) system. The effect of factors such as molecular weight of PEG, aging treatment, aging or gelation temperature, and the ratio of PEG to dioxane were investigated. The results revealed that all scaffolds had high porosity, and had a significant impact on the formation of nanofibrous structures. The decrease in the molecular weight and aging or gelation temperature leads to a thinner and more uniform fibrous structure.

## 1. Introduction

The aim of tissue engineering is to explore and develop a series of biological substitutes that are used to repair or replace damaged or diseased tissues. In a previous study reporting on the improvement in tissue repair and promotion of cell differentiation, researchers developed various scaffolds that had a structure similar to that of the tissue [1] [2]. Materials used to fabricate scaffolds should be non-toxic and exhibit a certain mechanical support performance. Additionally, they should exhibit a three-dimensional structure that is conducive to adsorption, transformation, cellular proliferation, and differentiation [3]. Scaffolds with nanofibrous structures have relatively great application prospects that are similar to those of naturally occurring extracellular matrix (ECM) [4–6].

Until now, several methods, such as electrospinning technology, thermal-induced phase separation technique (TIPS), 3D printing techniques have been used to prepare polymer scaffolds [7–11]. However, the disadvantages of electrospinning are the use of organic cytotoxic solvents and the small volumes of scaffolds obtained at low rates [12]. 3D printing processes have been developed in recent years that allow for spatial control of the composition of scaffolds at the micron scale [13]. The approach of TIPS is based on thermal energy changes that create a quench route separation of a homogenous polymer solution into a multi-phase device domain. The polymer is initially dissolved at a higher temperature to generate a homogenous solution in a solvent with a high boiling point and low molecular weight such as phenol or naphthalene, and biologically active molecules release into these solutions. The separation is caused during the temperature lowers and the homogeneous solution separates either by solid-liquid demixing or liquid – liquid phase separation mechanism into a polymer-rich phase and a solvent-rich phase. In order to generate a porous scaffold with bioactive compounds incorporated into the structure, the solvent is then removed by extraction, evaporation and sublimation. This approach can provide high porosity with macro and micro pores up to 90% [14]. The parameters affecting the scaffold structure such as polymer type, solvent, solution concentration, temperature, cooling rate, and additives can be easily regulated. Of these elements, the solution is the most relevant source to pore structure. The benefit of this technology is that the procedure is easy, it has a low faults rate, it achieves high porosity, and it easily mixes with other manufacturing methods [15]. Scaffolds fabricated using TIPS could facilitate the achievement of a three-dimensional network structure that is similar to that of naturally occurring ECM.

Poly (L-lactic acid) (PLLA) and Polycaprolactone (PCL), are common biodegradable synthetic polymers used for tissue engineering research [16–18]. These polymers have received increased attention due to their advantages, including ease of processing, adjustable degradation rate, and low inflammability. Specifically, PLLA has been widely used for bone, blood vessel, and cardiac tissue replacement [19–23]. However, PLLA has some disadvantages, such as the hydrophobicity of the material surface, the acidic nature of the degradation product, and the fact that the rate of degradation is not similar to the rate of bone regeneration [24]. Hydroxyapatite (HA) is an inorganic, weakly alkaline mineral that is an important part of the bone and teeth, and accounts for about 65% of the total weight in bone quality. Synthetic hydroxyapatite has a composition that is similar to that of naturally occurring hydroxyapatite. It has comparable bone conductibility, bone inductivity, and biocompatibility, and can bond directly with bone tissue during implantation. Various in-vitro and in-vivo experiments show that the surface of HA gathers more osteoblast [25]. However, HA has some disadvantages, such as brittleness and impact resistance. Therefore, HA is added to highly polymeric materials, such as gelatin, chitosan, PLLA, and PCL, to obtain composite materials [26–29]. For example, HA and PLLA composite scaffolds can not only improve the mechanical strength, compared to PLLA scaffolds, but also neutralize the acidity that occurs during the degradation of PLLA, thus effectively promoting the regrowth of bone tissues.

In this study, a series of PLLA and HA/PLLA scaffolds with the nanofibrous structure were fabricated using TIPS and a ternary PLLA/dioxane/polyethylene glycol system. Polyethylene glycol (PEG) is a low molecular weight, water-soluble polyether. Its hydroxyl value decreases as the freezing point and viscosity increase, resulting in an increase in its molecular weight. It has a long chain structure and is a poor solvent for PLLA. It is expected to increase the porosity of the scaffold if it is used to replace the undesirable solvent water in TIPS. The effects of the molecular weight of PEG, aging treatment, aging or gelation temperature, and the ratio of PEG to dioxane were investigated, and the optimal ratio of HA: PLLA was also examined.

## 2. Materials and methods

### 2.1 Materials

PLLA (M_n_ = 150,000) was purchased from Jiangsu Youli Technologies Ltd. All other reagents and solvents of analytical grade were purchased from Sinopharm Chemical Reagent Co.

### 2.2 Fabrication of nanofibrous PLLA scaffolds

The nanofibrous PLLA scaffolds were fabricated using the liquid-liquid phase separation technique in the PLLA/dioxane/PEG ternary system. First, 7% PLLA was prepared by dissolving PLLA in a dioxane/PEG solvent mixture with various mass ratios (95/5, 90/10, 85/15, and 80/20), at 60℃. Second, 5 mL of 7% of transparent PLLA solution was poured into a beaker mold (3.5 cm in diameter and 5 cm in height). Third, the samples were quenched using a predetermined temperature (*T*_*age*_ = 50, 40, 35, 30, 25, and 20℃) and aged for 2 h, followed by quenching at−15℃ (*T*_*gel*_) for 2 h to freeze the samples completely. The containers containing polymer solutions in the gel form were then transferred into ice-cold distilled water (4℃) to release the molds and to enable the exchange of solvent with H_2_O. The distilled water was replaced at least twice daily. Later, samples were frozen at−40℃ for 1 h and lyophilized for 4 d to obtain the scaffolds. All the dried scaffolds were trimmed and placed in a desiccator prior to their use.

### 2.3 Fabrication of HA/PLLA scaffolds

First HA/PLLA scaffolds were prepared by dissolving 7% PLLA and varied amounts of HA (m_HA_/(m_HA_+m_PLLA_) of 2%, 5%, 10% and 20%) in dioxane/PEG2000 or a PEG200 solvent mixture with a mass ratio of 95/5 at 60℃ using ultrasonic oscillation for 30 minutes. Second 5 mL of transparent HA/PLLA polymer solution was poured into a beaker mold, quenched at 25℃, and aged for 2 h (this step is not applicable for PEG200), followed by quenching at−15℃ (*T*_*gel*_) for 2 h to freeze the samples completely. The remaining steps were similar to those used for the fabrication of PLLA scaffolds.

### 2.4 Characterization of scaffolds

The morphology of the scaffold was observed by scanning electron microscopy (SEM, Japan JSM-7500F). To expose the internal and cross-sectional structure, a sample was fractured after being frozen in liquid nitrogen for several minutes. The scaffold sample was coated with gold by a sputter coater, and was analyzed at 5 kV. The water contact angle was measured using a model SL200 Contact Angle Meter. FTIR spectra were measured to confirm the formation of nanofibrous PLLA scaffolds. Avatar 360 spectrometers, with KBr pastille, was used for FTIR characterization. The analysis range was from wave number 4000 to 400 cm^−1^. DSC was used to analyze the thermal properties, Metter Toledo 822e in a temperature range from 20℃ to 200℃ with the heating rate of 10℃/min. XRD was used to analyze the crystal structure of scaffolds by a Philips X’pert MPD X diffractometer using Cu Ka generated at 40 KV and 40 mA. The samples were scanned from 10° to 80° with a step size of 0.02° and a count rate of 3.0°/min. The porosity of the scaffolds was determined by a simple method [30,31]. CCK-8 assay was used to assess cell viability. Briefly, CCK-8 (Beyotime, China) was added into the culture medium and incubated at 37℃ for 2 h. The ratio of medium: CCK-8 was 10:1 by volume. The optical density of the cell suspension was measured at 450 nm (OD_450 nm_) using a microplate spectrophotometer. All the data are expressed as means ± standard deviation (SD), and a *p*-value<0.05 was considered statistically significant.

## 3. Results and discussion

### 3.1 The influence of different solvent systems on the morphologies of PLLA scaffolds

**Figure 1.**
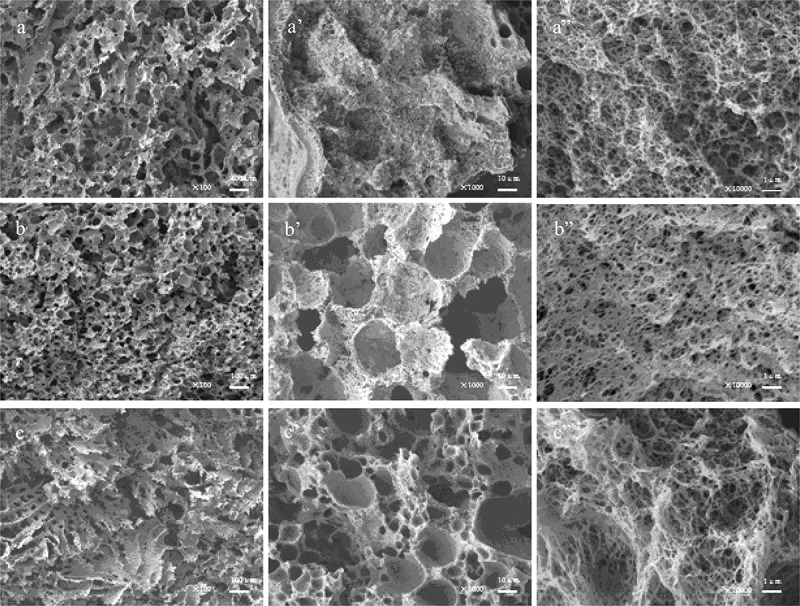
SEM diagrams of PLLA scaffolds fabricated by the different solvent systems (the solvent systems were 1,4-dioxane/water(a,a’,a”), 1,4-dioxane/peg200(b,b’,b”), 1,4-dioxane/peg2000(c,c’,c”), respectively).

The morphologic evolution of PLLA scaffolds in different solvent systems has been presented in Figure 1. The scaffolds were fabricated using 7 wt% PLLA solution in 95/5 (V/V) dioxane/water, dioxane/PEG200, and dioxane/PEG2000, were referred to as PLLA0, PLLA200, and PLLA2000 respectively. As shown in Figure 1, the macro/micropores in both PLLA200 and PLLA2000 were increased as compared to PLLA0. An increase in the molecular weight of PEG was accompanied by an increase in the size of macropores. Pore size is one of the signifciant characteristics of scaffolds for bone tissue engineering. Based on early studies, scaffolds need approximately 100 μm of the minimum pore size because of cell size, transport, and migration requirements [32,33].

**Figure 2.**
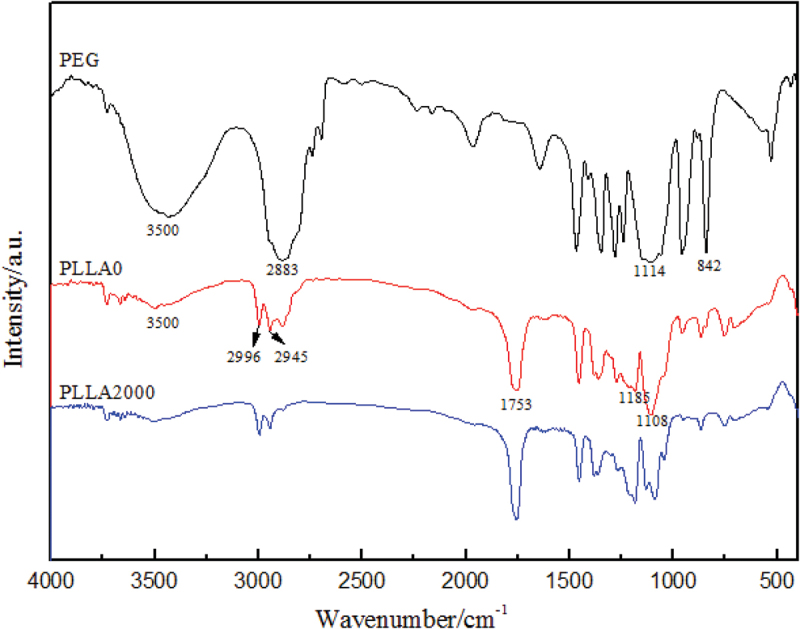
FTIR diagrams of PEG, PLLA0 and PLLA2000.

In Figure 2 the FTIR spectrum of PEG (a), PLLA0 (b), and PLLA2000 (c) have been shown. The strong adsorption at 3500 cm^−1^ is attributable to the stretching vibration of the – OH group structure. The peaks corresponding to 2996, 2945 and 2883 cm^−1^ were assigned to the stretching vibration of the saturated C-H group. The stretching vibration at 1114 cm^−1^ was attributable to the saturated C-O group, the swing vibration at 842 cm^−1^ was attributed to the – CH_2_- group, and the peak at 1753 cm^−1^ was attributable to the stretching vibration of C=O bond. These facts, clearly indicate that PEG does not remain in the scaffold and it acts as the non-solvent in the ternary system.

### 3.2 The influence of the molecular weight of PEG on the morphologies of PLLA scaffolds

**Figure 3.**
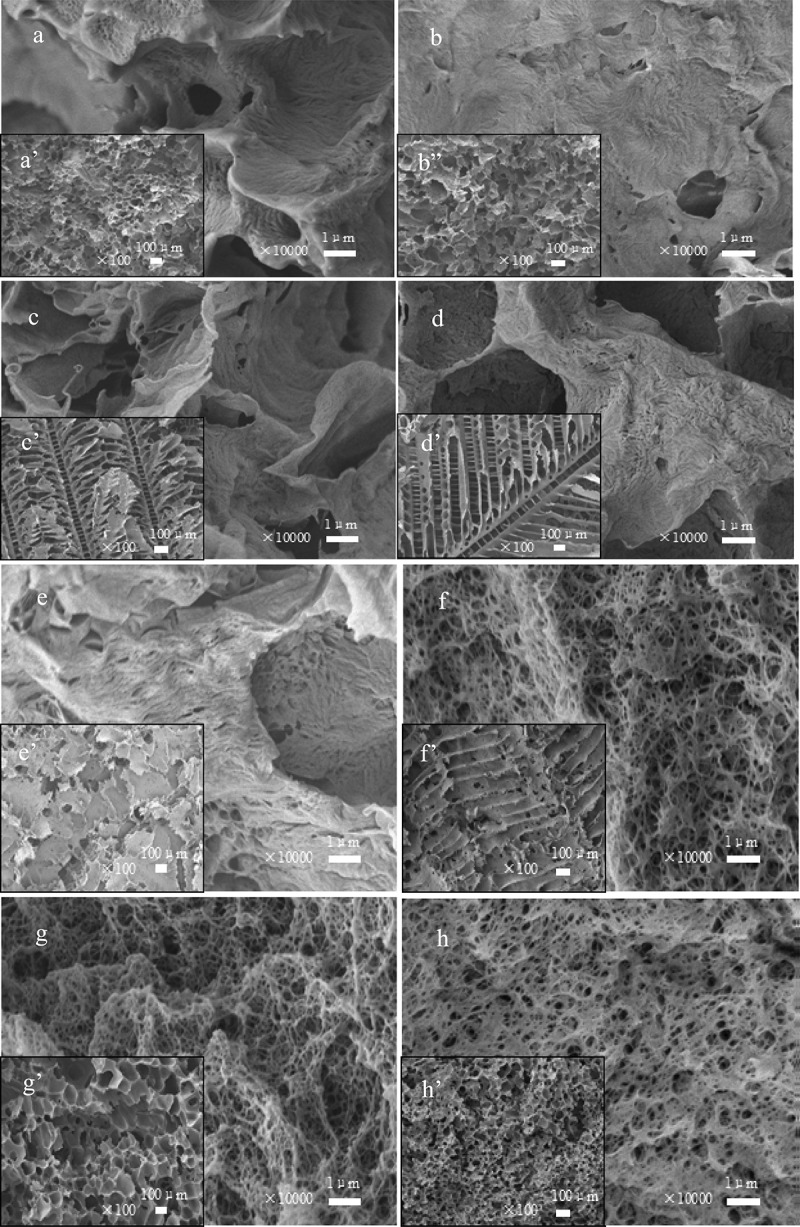
SEM diagrams of PLLA scaffolds fabricated by the different molecular weight of PEG (the molecular weight of PEG were 10,000(a, a′), 6000(b, b′), 4000(c, c′), 2000(d, d′), 1500(e, e′), 600(f, f′), 400(g, g′), 200(h, h′), respectively).

The molecular weight of PEG was an important factor that influences the morphology of PLLA scaffolds. Figure 3 illustrates the morphologies of PLLA scaffolds prepared using different molecular weights of PEG at−15℃, using a gel that is not subjected to aging. As the molecular weight of PEG in the solvent system gradually decreases, the tendency for the formation of nanofibers in the scaffolds becomes more apparent. When the molecular weight of PEG was between 10,000 and 1500, the freezing points were observed to be in the range of 40℃ to 63℃. The polymer solution underwent cold crystallization and nucleation to form a molecular structure at the interface of the solid wall. Besides, the long-chain structure of PEG induced crystals separates out from the orientation of the polymer scaffolds, thus, leading to PLLA scaffolds with a certain configuration (Figure 3A’, B’, C’, D’, E’) [34]. As the molecular weight of PEG decreases from 600 to 200, the freezing point was observed to be below 20℃. The non-solvent system was closer to other commonly used liquid non-solvents, such as water and ethanol. Thus, it was easier to form a relatively uniform nanofibrous structure (Figures 3F, G, and H).

### 3.3 The influence of aging on the morphologies and crystallization of PLLA scaffolds

**Figure 4.**
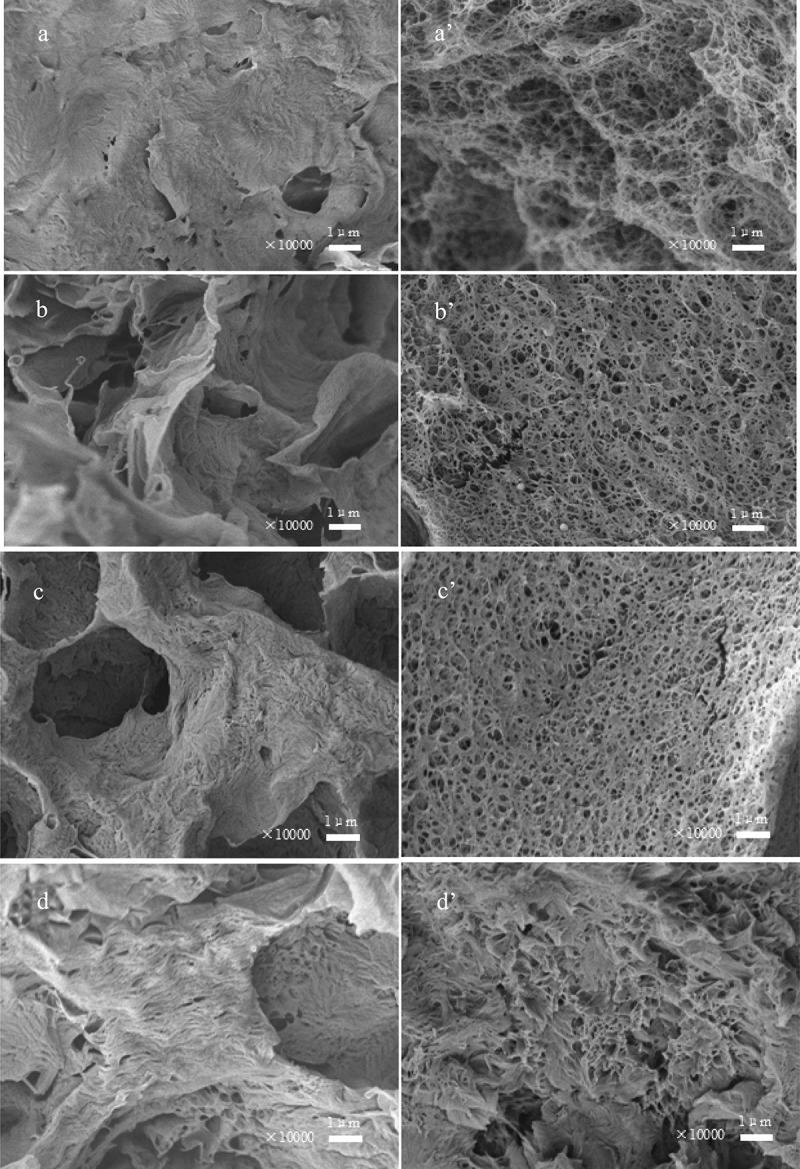
SEM diagrams of PLLA scaffolds fabricated without/with aging by the different molecular weight of PEG (the molecular weight of PEG were 6000(a, a′), 4000(b, b′), 2000(c, c′), 1500(d, d′), respectively).

Aging influences the formation of nanofiber to a certain extent. The SEM images of PLLA scaffolds fabricated without or with aging using dioxane/PEG solvent system with different molecular weights of PEG are shown in Figure 4. Although scaffolds in the gel that has not been subjected to aging at−15℃ had an interconnected structure, the surface exhibited a spherulitic crystal structure. However, all scaffolds aged at 30℃ for 2 h before gel formation at−15℃ had formed a uniform nanofibrous network structure.

**Figure 5.**
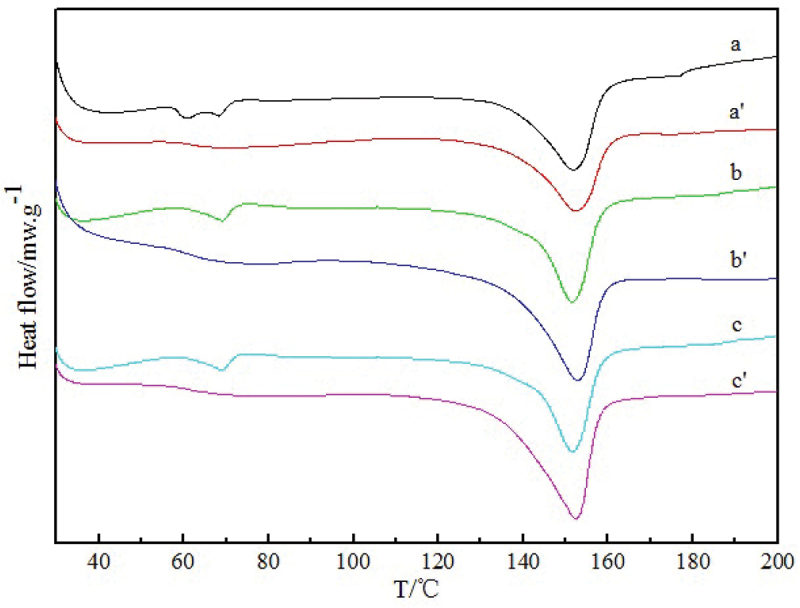
DSC thermogram of PLLA scaffolds fabricated without/with aging by the different molecular weight of PEG (6000 (a, a′), 4000 (b, b′), 2000(c, c′), respectively).

DSC analysis as shown in Figure 5 was performed to study the crystallization behaviors of PLLA. The degree of crystallinity (χ_c_) was calculated and the results were found to be more optimal for specimens aged in the gel state than those obtained without the aging process (Table 1).

**Table 1.** The degree of crystallinity (χ_c_) of PLLA scaffold prepared in 7% (w/w) dioxane/water = 95/5 (w/w) with/without aging in the gel status at−15℃.

	Without aging	Aging for 2 h
PLLA 6000	13.96%	17.96%
PLLA 4000	17.23%	24.91%
PLLA 2000	19.66%	30.29%

The phase separation of a polymer solution can also be considered as a self-assembly process. The ease of aggregation of large molecules in a new phase using an initial homogenous one-phase system is greater than that achieved using smaller molecules. Aging in the gel state was essential for the formation of the nanofibrous network. During this process, PLLA chains would rearrange and further crystallizes. The crystallization of PLLA in the gel would be conducive to the initiation of the phase separation process [35].

### 3.4 The influence of aging temperature (T_a_) and gelation temperature (T_gel_) on the morphologies and crystallization of PLLA scaffolds

**Figure 6.**
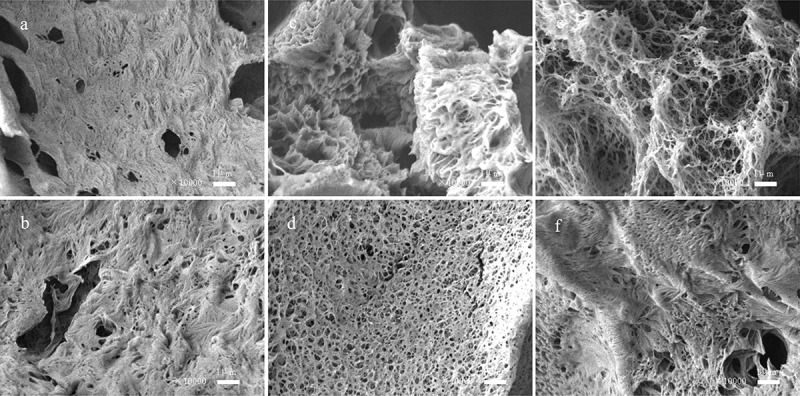
SEM diagrams of PLLA scaffolds fabricated by PEG2000 with different T_a_ (T_a_ = 50℃(a), 40℃(b), 35℃(c), 30℃(d), 25℃(e), 20℃(f), respectively).

Figure 6 displayed the morphologic evolution of PLLA scaffolds as a function of *T*_*a*_. These scaffolds were fabricated from 7 wt% of PLLA solution in 95/5 (w/w) dioxane/PEG2000 at different *T*_*a*_ values (50℃, 40℃, 35℃, 30℃, 25℃, 20℃) for 2 h at−15℃. The characteristic ratios of macro/micropores were observed in all matrixes. At a higher *T*_*a*_ (Figure 6a and b), the scaffold was interconnected, however, the nanofibrous structure was found to be compact. Notably, as the T_a_ decreases gradually, the diameter of fiber was decreased simultaneously.

**Figure 7.**
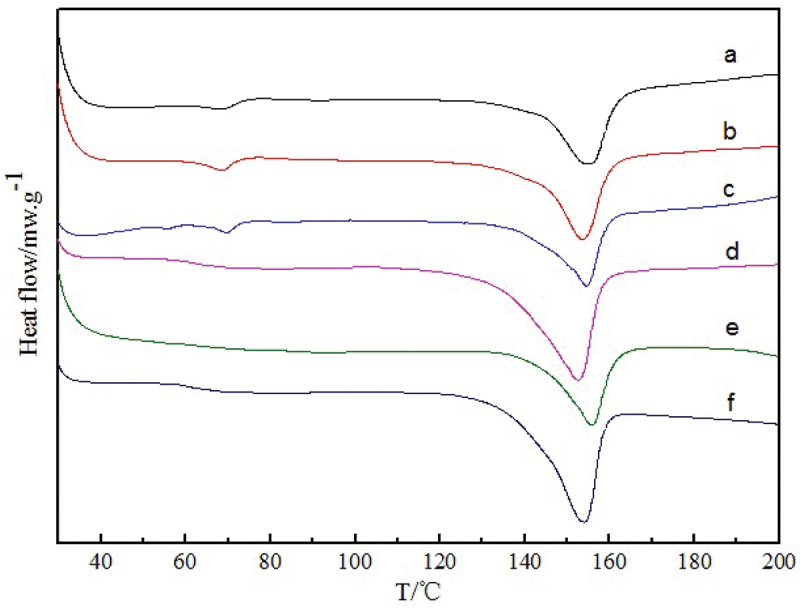
DSC thermogram of PLLA scaffolds fabricated by PEG2000 (95/5) with different T_a_ = (a)50℃, (b)40℃, (c)35℃, (d)30℃, (e)25℃, (f)20℃.

The thermogram of PLLA analyzed by DSC has been presented in Figure 7. As shown in Table 2, the degree of crystallinity increased as the temperature decreases, leading to the formation of more uniform nanofibers, this could be attributed to the rearrangement of the PLLA chain [36].

**Table 2.** The degree of crystallinity (χ_c_) of PLLA scaffold prepared in 7% (w/w) dioxane/water = 95/5 (w/w) with different T_a_ in the gel status at−15℃.

Aging temperature (T_a_)/℃	The degree of crystallinity (χ_c_)
50	16.28%
40	19.11%
35	20.26%
30	30.29%
25	24.91%
20	21.94%

**Figure 8.**
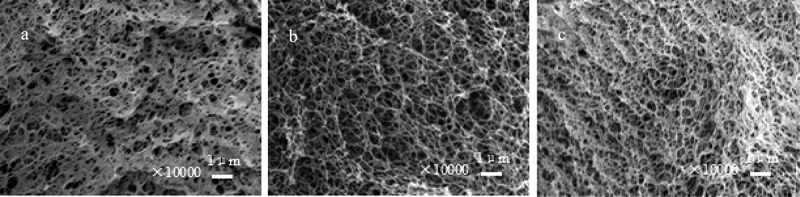
SEM diagrams of PLLA scaffolds fabricated by PEG200 with different *T*_*gel*_ (*T*_*gel*_ was−15 ℃ (a), −25 ℃ (b), −40 ℃ (c), respectively).

The gelation of the PLLA/dioxane/PEG200 ternary system played a crucial role in creating a unique nanofibrous structure. The morphologies of PLLA scaffolds prepared with different *T*_*gel*_ have been shown in Figure 8. When *T*_*gel*_ was below−15℃, the polymer solution was able to form the scaffold with a uniform nanofibrous structure. As *T*_*gel*_ decreased, both the diameter of the fiber and the size of the pore decreased.

The morphology of scaffolds was found to have a strong dependence, which was in agreement with the dependence of crystallinity degree [37]. Similar to the PLLA/dioxane/water system, a low *T*_*a*_ or *T*_*gel*_ value would lead to the formation of nanofibrous structures. Ma et al hypothesized that the formation of the nanofibrous structure was related not only to the liquid-liquid phase separation but also to the consequential crystallization of the polymer-rich phase [38]. A Rapid cooling rate was needed to avoid polymer crystal nucleation and growth, in order to obtain a uniform nanofibrous network structure. Meanwhile, the crystallization of the solvents with a high freezing point would further limit the polymer crystal, thus, the fibrous structure was obtained easier when the *T*_*a*_ or *T*_*gel*_ was lower.

### 3.5 The influence of the ratio of dioxane/PEG on the morphologies of PLLA scaffolds

**Figure 9.**
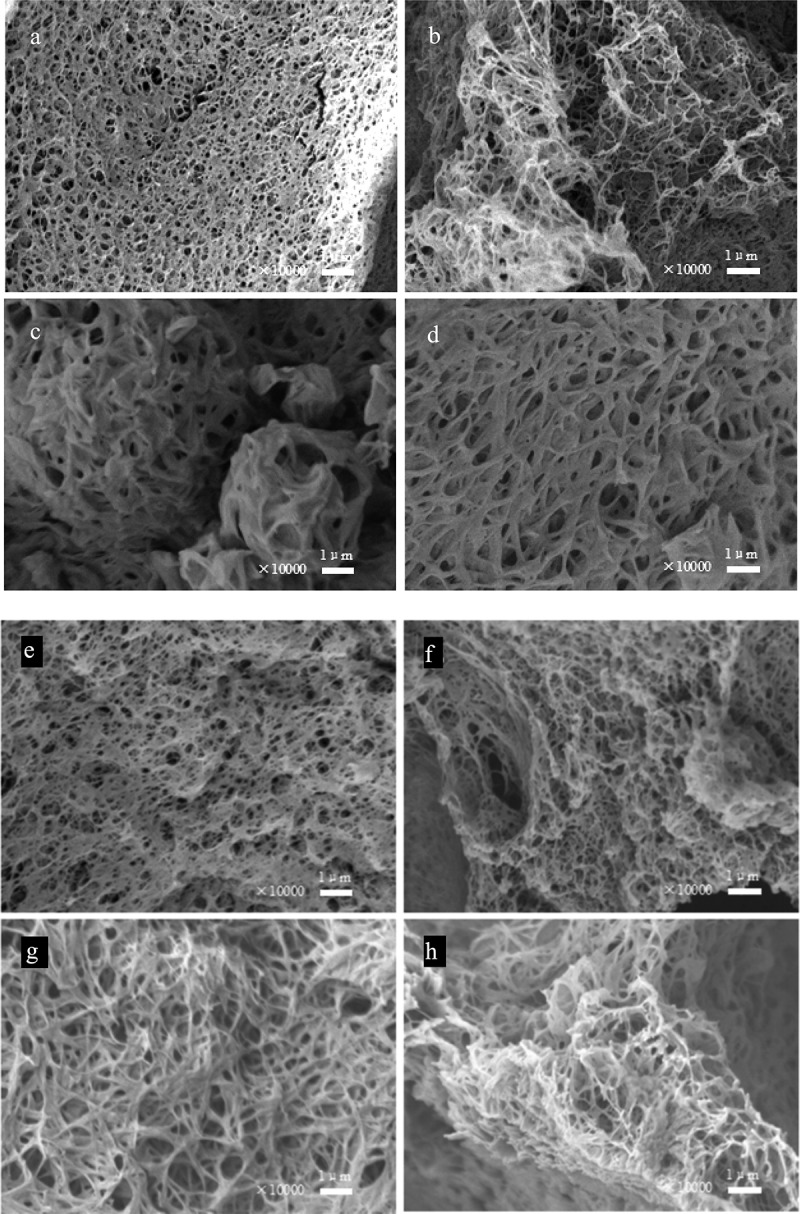
SEM diagrams of PLLA scaffolds fabricated with different rate of solvent system. dioxane/PEG2000 = 95/5(a), 90/10(b), 85/15(c), 80/20(d),and dioxane/PEG200 = 95/5(e), 90/10(f), 85/15(g), 80/20(h).

The ratio of dioxane/PEG was another influential factor that affected the morphology of the scaffolds. As shown in Figure 9, the formation of non-homogenous pores with a large size was observed as the proportion of PEG in the solvent mixture was increased. Porosity test results have been listed in Table 3. The porosity decreases with an increase in the proportion of PEG in the solvent mixture. This might be due to the poor solubility of PEG which was observed with an increase in the ratio of non-solvent liquids. This is because when the polymer is dissolved in the solvent, the addition of the non-solvent is equivalent to dilution of the polymer solution, which makes the polymer system more inhomogeneous, and the viscosity reduction of the co-solvent system induces the formation of larger droplet areas in the polymer-poor phase, so the pore size has increased [16].

**Table 3.** Porosity of PLLA scaffolds under different ratio of dioxane/PEG200.

The ratio of dioxane/PEG	Porosity of scaffold
95/5	93.18%
90/10	92.13%
85/15	91.47%
80/20	89.89%

### 3.6 The influence of adding HA into the polymer system on the morphologies and crystallization of PLLA scaffolds

**Figure 10.**
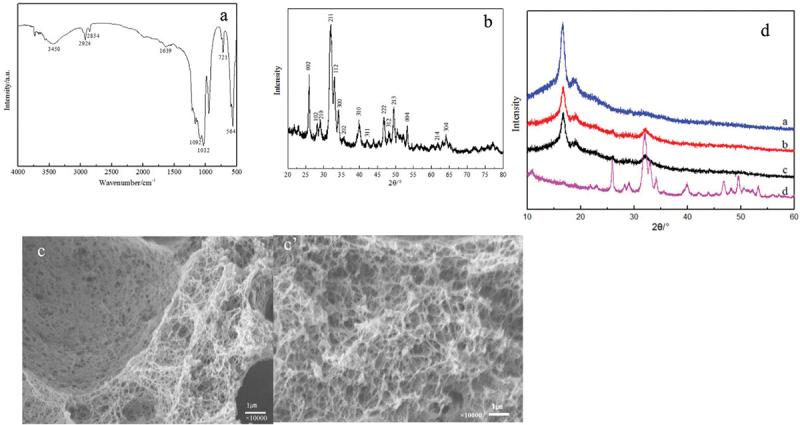
FTIR (a) and XRD (b) of the HA sample. SEM of scaffolds morphology before and after adding 5% HA, PLLA200 (c) and PLLA2000 (c′). XRD of different scaffolds (a) PLLA, (b) HA/PLLA 2000, (c) HA/PLLA 200, (d) HA.

In order to promote the regrowth of bone tissue, a certain amount of HA prepared and tested by FTIR and XRD (Figures 10a, 10b), was added to the polymer to fabricate HA/PLLA composite scaffolds. The morphologies of PLLA scaffolds prepared using 5 wt% HA in PLLA200 and PLLA2000, have been displayed in Figure 10c and d, respectively. The composite scaffolds maintained the nanofibrous structure with HA adhesion at the points of contact of the fibers. Additionally, its porous structure was maintained. Figure 10E was the XRD pattern of scaffolds before and after the addition of 5 wt% HA. The main diffraction peaks of PLLA were observed both at 16.7° and 19.1°, however, there were two more diffraction peaks observed at 25.9° and 32° (curve b and c), which was attributed to the addition of HA. In addition, the half peak width and the diffraction peak intensity of the composite scaffolds were decreased slightly. This might be attributable to the presence of the heterogeneous nucleating agent after the addition of HA into the polymer solution, and it affected the movement of the PLLA molecular chain, thus decreasing its crystallinity [39].

**Figure 11.**
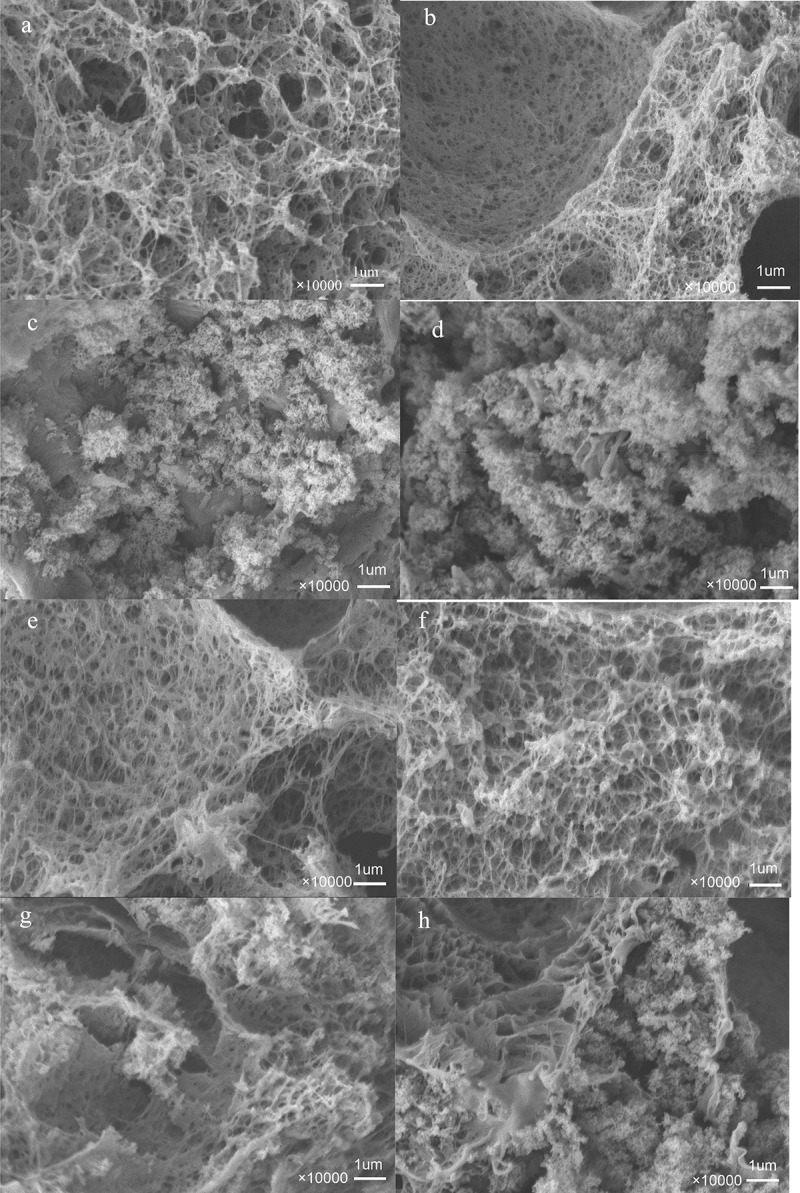
SEM of PLLA200 scaffolds (abcd) and PLLA2000 scaffolds (efgh) with different rate of HA: 2% (ae), 5%(bf), 10%(cg), 20%(dh).

**Figure 12.**
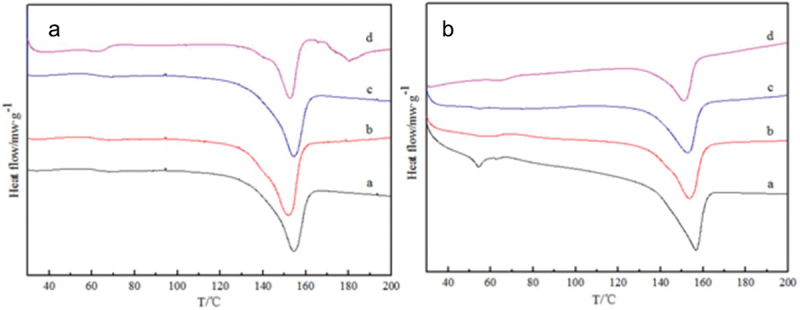
DSC thermogram of HA/PLLA200 scaffolds (a) and HA/PLLA2000 scaffolds (b) with different proportion of HA (a.2%, b.5%, c.10%, d.20%).

The SEM image with different proportions of HA added into the polymer solution has been shown in Figure 11. When HA was mixed in a proportion of 2 wt% or 5 wt%, the powder could spread uniformly in the composite scaffold. In addition, the clarity of the images was enhanced by the increased addition of HA to the polymer, the proportion of HA added was above 10 wt%. DSC thermograms of these composite scaffolds were shown in Figure 12, and the transformation of PLLA thermal performance and crystallinity has been displayed in Table 4. The introduction of HA affected the shape and temperature of the melting peak to a lower extent, however, the enthalpy and crystallinity were found to increase at first, and then these values decrease. As HA occurred in the form of a nanopowder it resulted in the formation of crystalline nucleus in the mixed system, which had some effects on the crystallization of PLLA. The structure was affected because of the continuing increase in the levels of HA, which might prove to inhibit the movement of the PLLA molecular chain [40]. After taking the structure and the proportion of HA into consideration, we concluded that the best proportion of HA required for the fabrication of the composite scaffolds was 5 wt%.

**Table 4.** The transformation of PLLA thermal performance and crystallinity.

Sample name		Melting temperature(℃)	Melting enthalpy(J·g^−1^)	Crystallinity
HA/PLLA200	2%	153.83	22.95	24.52%
	5%	152.02	25.77	27.53%
	10%	154.30	23.88	25.51%
	20%	152.91	15.89	16.97%
HA/PLLA2000	2%	154.63	22.54	24.08%
	5%	153.90	26.96	28.80%
	10%	153.07	20.23	21.61%
	20%	152.34	14.34	15.32%

### 3.7 Cytocompatibility Evaluation

**Figure 13.**
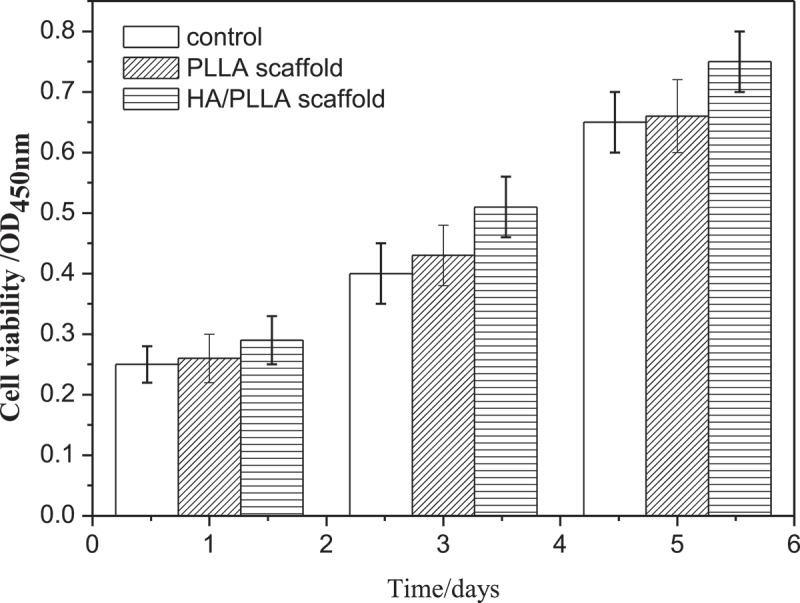
Cell viability of MC3T3-E1 cells cultured on different substrates after 5 days of incubation.

As shown in Figure 13, there was no significant difference in cell survival rate on the surface of PLLA scaffold and control group after 1 day of cell incubation, whereas cell survival rate of HA/PLLA scaffold was increased. When the incubation time was extended to 5 days, HA/PLLA scaffold showed higher cell activity than the control group, indicating that HA/PLLA scaffold has good cytocompatibility.

## 4. Conclusions

In this study, nanofibrous PLLA and HA/PLLA scaffolds were fabricated using the TIPS technique from a ternary PLLA/dioxane/PEG system. The PLLA scaffolds were obtained by controlling the molecular weight of PEG, *T*_*a*_ or *T*_*gel*_, and the ratio of dioxane/PEG. The nanofibrous structure was found to occur while the molecular weight of PEG decreased, otherwise, the nanofibrous structure occurred when *T*_*a*_ or *T*_*gel*_, values were lowered. Moreover, the crystallization of PLLA had a substantial effect on the fabrication of nanofiber if the crystallization occurred to a lesser extent the nanofibrous structure could not be formed. The optimal level of hydroxyapatite that needed to be added to the polymer system in order to enhance the formation of newborn bone tissue was 5 wt%, and hydrophilicity was observed to improve simultaneously.

## Data Availability

The data that support the findings of this study are available from the corresponding author, upon reasonable request.
